# Translational Potential of RNA Derived From Extracellular Vesicles in Multiple Myeloma

**DOI:** 10.3389/fonc.2021.718502

**Published:** 2021-08-27

**Authors:** Antonia Reale, Tiffany Khong, Sridurga Mithraprabhu, Andrew Spencer

**Affiliations:** ^1^Myeloma Research Group, Australian Centre for Blood Diseases, Central Clinical School, Monash University/Alfred Health, Melbourne, VIC, Australia; ^2^Malignant Haematology and Stem Cell Transplantation, The Alfred Hospital, and Department of Clinical Haematology, Monash University, Melbourne, VIC, Australia

**Keywords:** extracellular vesicles, RNA, myeloma, tumour microenvironment, translational medicine, biomarker, liquid biopsy

## Abstract

The cross-talk between tumour cells and stromal cells is a hallmark of multiple myeloma (MM), a blood cancer that still remains incurable despite increased knowledge of its biology and advances in its treatment. Extracellular vesicles (EVs) derived from both tumour and stromal cells have been shown to play an important role in mediating this cross-talk ultimately favouring MM progression and drug resistance. Furthermore, EVs and their content including RNA (EV-RNA) have been successfully isolated from blood and are being explored as liquid biomarkers in MM with the potential to improve diagnosis and monitoring modalities with a minimally-invasive and repeatable analysis, i.e. liquid biopsy. In this review, we describe both the role of EV-RNA in defining the biological features of MM and their potential translational relevance as liquid biomarkers, therapeutic targets and delivery systems. We also discuss the limitations and technical challenges related to the isolation and characterization of EVs and provide a perspective on the future of MM-derived EV-RNA in translational research.

## Introduction

Multiple myeloma (MM) is a blood cancer characterised by the clonal expansion of malignant plasma cells (PCs) within the bone marrow (BM) (1). While, a better understanding of MM biology and the introduction of novel drugs and treatment options have provided an improved framework for the management of MM patients resulting in increased duration of survival (2–5), MM remains incurable with a median survival of 5 years, and only 2 years for high-risk patients (2). This variation in outcome is mirrored by the highly (genetically) heterogeneous nature of the disease, both spatially and temporally (6) with multiple foci of disease at diagnosis containing sub-clones that evolve genetically over time contributing to drug resistance (3). Recently, analyses of body fluids including blood (i.e. liquid biopsies) have generated significant interest due to their potential for the characterisation of tumours with a rapid and non-invasive procedure (7–18). In the context of MM, accumulating evidence suggest that liquid biopsy captures the genetic heterogeneity of the disease with important implications for circulating tumour DNA (ctDNA) and cell-free or extracellular RNA (exRNA) as valuable markers for tumour genome characterisation, prognostication and sequential monitoring of disease (6–13). For these reasons liquid biopsies hold promise as an alternative or addition to single-site BM biopsies that are invasive and fail to capture MM tumour heterogeneity (19).

EVs (small EVs or exosomes ~30-150nm in diameter; large EVs or shed microvesicles 200 nm to ~1,300 nm), particles delimited by a lipid bilayer (20), are released from all cell types into the extracellular space and play key roles in many physiological and pathological processes including cancer (20, 21). Several recent studies have addressed the potential functions of EVs, both *in vitro* and *in vivo*, and have defined characteristic properties, with the aid of ‘omic’ profiling (16, 17, 22–27).

It is well-established that cells actively incorporate molecules (proteins, lipids, nucleic acids) into EVs which may be transferred to target (recipient) cells making EVs an important means of cell-to-cell communication both at local and distant sites (28, 29). As such, EVs are able to modulate the function of target cells by reprogramming signalling pathways, and in a cancer context, promote the formation of a supportive tumour microenvironment (TME) and pre-metastatic niches (28, 29). Furthermore, EVs share common surface markers with their cell of origin, designating them as a promising target for biomarker discovery, diagnostics and therapeutics in cancer (14, 28, 30). Importantly, EVs protect their content (cargo) including RNAs from degradation in the extracellular environment and can be successfully collected for downstream analyses from biofluids including blood, making them ideal candidates for liquid biopsy (14, 30, 31). In this review, we discuss the current knowledge on the role of EVs in MM with a particular focus on EV-RNA and their potential translational application in MM.

## EVs and Their Characterization

EVs can be isolated and purified from both biofluids and cell culture supernatants (20, 31, 32) although a consensus on the optimal source material (i.e. plasma vs serum) and the standardization of pre-analytical variables and reporting are still lacking. Their isolation can be challenging due to their biophysical properties and the presence of other factors which are often co-isolated, including highly abundant proteins (e.g. albumin), impairing both the enrichment of highly purified EVs and downstream applications (e.g. genomics, proteomics - ’omics’) (27, 33–36). We have recently demonstrated, for the first time in MM, that the depletion of albumin in plasma-derived small EVs for mass-spectrometry based proteomics can improve protein detection (17, 31). Several protocols based on different methods have been developed for successful EV isolation from different source materials (31, 37–39). Regular position statements are published to provide the scientific community with protocols for EVs isolation, purification, analyses, accurate reporting of pre-analytical variables (e.g. use of plasma versus serum, processing times, storage/thawing conditions) and methodologies (20, 40–42).

Highly sensitive and selective omics technologies with the aid of advanced bioinformatic tools, have been successfully utilized for EV-cargo characterization and biomarker discovery in MM with most studies focusing on RNA-based downstream applications (7, 8, 31, 43).

### EV-RNA

The RNA content of EVs varies depending on the source material (e.g. type of biofluid, cell of origin and its physiological or pathological state) and the EV subpopulation but with differences also within the same EV subtype (32). The loading of RNAs into EVs appears to be a tightly regulated process involving both active or passive mechanisms which largely depend on RNA binding proteins (RBPs) and their partners as well as RNA motifs and modifications (44, 45).

Messenger RNAs (mRNA), most likely fragmented, have been widely reported as EV cargo together with non-coding RNAs including Y-RNA, tRNA-derived small RNA fragments (tDRs), long non-coding RNAs (lncRNA), micro-RNAs (miRNA) (46). Publicly available databases provide comprehensive EV-RNA profiles, e.g. Extracellular RNA Communication Consortium (ERCC) (42), exoRBase (47), exoCarta (48), Vesiclepedia (49), exRNa Atlas (50), EVmiRNA (51).

miRNA are the most studied and characterized extracellular RNA subtype contained within the EV-cargo (46, 52). Hundreds of miRNA species can be detected in a given biofluid with increasing evidence that each biofluid has a distinct miRNA composition. miRNA are small non-coding RNAs of ~20-25 nucleotides in length that can modulate gene expression by mRNA silencing and translational activation, and exhibit tissue- and state-specific expression patterns. EV-miRNA can be transferred to recipient cells resulting in phenotypic and functional changes and as such are constituents of tumour-derived EV-cargoes with potentially important functions in the context of the tumour biology (e.g. migration, invasion, proliferation) including MM (44, 53–55). EV-miRNA from tumour cells can also reprogram stromal cells and immune cells to support their growth, invasion, metastasis and drug resistance (44, 54). Despite the technical constraints, small RNA sequencing of circulating small EVs has revealed that miRNA is the predominant species of RNA present in MM-small EVs, while small nuclear and nucleolar RNA, ribosomal RNA, lncRNA, and unclassified RNA represent a smaller percentage (8).

## Biological Roles of EV-RNA in MM

**Figure 1:**
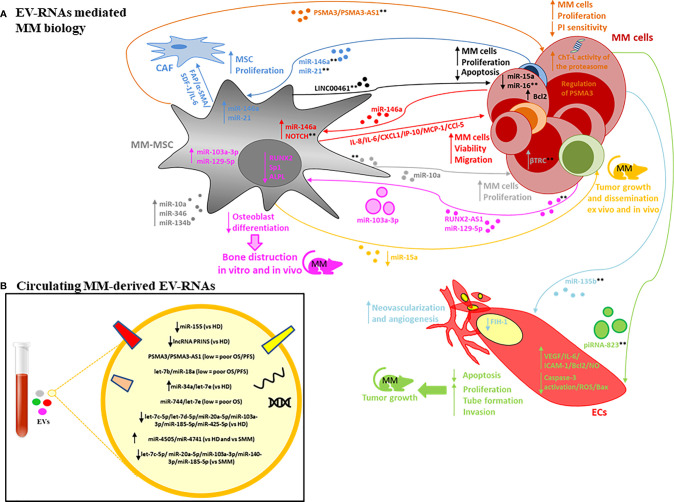
**(A)** EV-RNAs involved in MM pathobiology. The RNA content of MM-derived EVs is involved in mesenchymal stromal cells (MSC) proliferation and cancer-associated fibroblasts (CAF) transformation (dark-blue lines); neovascularization and angiogenesis, promoting endothelial cell (ECs) proliferation, invasion, reduced apoptosis and the release of angiogenic factors (light-blue and green lines); bone disease by altering osteoblast differentiation (pink line). Finally, MM-derived EV-RNAs enhance tumour cell viability and migration by altering NOTCH pathway and miR-146a levels in MSC with increased secretion of factors such as IL-6, CCl-5, MCP-1 (red lines). The RNA content of MSC-derived EVs is involved in tumour cell proliferation, apoptosis, dissemination, proteasome inhibitors (PI) sensitivity (orange, black, grey, yellow lines). Both large EVs and small EVs are depicted. **indicate possible targets based on use of specific inhibitors, gain or loss of function studies, and EV-secretion inhibitors (see text for description). **(B)** EV-RNAs identified within the cargo of circulating EVs isolated from patients with MM, MGUS, SMM *vs* HD.

It is well-established that the BM microenvironment (BMME) plays an important role in MM pathogenesis, including malignant transformation of the pre-malignant stages (monoclonal gammopathy of undetermined significance, MGUS; and smouldering MM, SMM) into active MM, and its resistance to drugs (56–59). Several pathological alterations within the BMME (e.g. angiogenesis, osteoclast activation, immunosuppression, osteoblast inhibition) promote proliferation, migration and drug resistance of mutated PC as well as decreased apoptosis and DNA repair function (56, 57). Cross-talk between PCs and the stroma is mediated by direct cell to-cell contact and soluble factors including cytokines, growth factors and EVs (16, 17, 60). EV-RNA derived from both the PC and stromal compartments has been shown (55, 61, 62) to contribute to the complications of MM and treatment failure *via* the promotion of angiogenesis, osteolysis and drug resistance, suggesting that the targeting of the EV-mediated cross talk may represent a novel therapeutic approach in MM. A schematic representation of EV-RNA mediated cell-to-cell interactions between MM cells and stromal cells, including molecular and functional changes in both compartments is shown in **Figure 1A**. The proposed RNA cargo of circulating MM-EVs is also depicted (**Figure 1B**). **Table 1** summarizes the publicly available data on EV-RNAs as mediators of MM progression and as potential biomarkers or therapeutic targets.

**Table 1:** Summary of findings on the role of EV-RNAs in MM pathobiology and as clinical biomarkers.

Expected EVs types	EVs isolation method	EVs source/donor cells (starting volume if applicable)	Target (recepient) Cells	Identified RNAs	Molecular effect in recepient cells	Biological effect in recepient cells	Expression levels (MM/MGUS *vs.* healthy controls)	Prognostic significance (biomarkers)	Potential therapeutic targets and specific inhibitors, gain or loss of function method	Reference
Small EVs	Ultracentrifugation	HMCL	Primary HD-MSC	miR-21; miR-146a	Overexpression of miR-21, miR-146a, FAP, α-SMA, SDF-1. Increased secretion of IL-6	MSC: Increased cell proliferation and CAF transformation			miR-21 or miR-146a (transfection with MiR-21/146a inhibitors)	(63)
Small EVs	Ultracentrifugation	Serum		miR-155			Low in MM *vs.* HD			(64)
Small EVs	Ultracentrifugation	HMCL; plasma from BM aspirates of MM/SMM patients	Human MSC	miR-129-5p	Downregulation of Sp1 and ALPL	Reduced osteoblast differentiation	High in MM *vs.* SMM			(65)
Small EVs	Ultracentrifugation +/- Precipitation (ExoQuick solution, System Biosciences)	BM-MSC from HD and MM patients; HS5 cells	HMCL	miR-15a		Reduced cell proliferation in vitro when treated with small EVs isolated from HS5 cells or BM-MSC derived from HD. Increased tumor growth and dissemination ex vivo and in vivo in the presence of MM BM-MSC–derived small EVs.	Low in small EVs isolated from BM-MSC derived from MM *vs.* HD			(66)
Small EVs	Precipitation (ExoQuick solution, System Biosciences)	Hypoxic HMCL	HUVEC	miR-135b	Downregulation of FIH-1	Endotelial tube formation (neovascularization) and angiogenesis			Anti–miR-135b inhibitor	(67)
Small EVs	Precipitation (ExoQuick solution, System Biosciences)	HMCL	Primary HD-MSC	miR-146a	Overexpression of miR-146a and secretion of several cytokines and chemokines including CXCL1, IL6, IL-8, IP-10, MCP-1, CCL-5. Involvement of NOTCH signaling.	HMCL: Increased cell viability and migration			NOTCH pathway inhibitor (DAPT)	(68)
Small EVs	Precipitation (ExoQuick solution, System Biosciences)	HMCL	Primary BM-MSC	lncRUNX2-AS1	Reduced expression of RUNX2	Reduced osteoblast differentiation			EV-secretion inhibitor GW4869	(69)
Small EVs	Precipitation (ExoQuick solution, System Biosciences)	BM-MSC from HD and MM patients	HMCL	miR-10a; miR-346; miR-135b		Increased cell proliferation	High in BM-MSC derived from MM *vs.* HD		βTRC, direct target of miR-10a (βTRC inhibitor). EV-secretion inhibitors (FTY720).	(70)
Small EVs	Precipitation (ExoQuick solution, System Biosciences)	Serum (0.25 mL)		miR-744; miR-130a; let-7d; let-7e/miR-34a			Low/High (Note: higher miR-34a and lower let-7d in RRMM *vs.* NDMM)	OS and TTP (miR-744; let-7e)		(71)
Small EVs	Precipitation (ExoQuick solution, System Biosciences)	Serum (0.5 mL)		let-7b; miR-18a			Low (MM *vs.* HD)	PFS and OS		(8)
Small EVs	Precipitation (ExoQuick solution, System Biosciences)	Serum		let‐7c‐5p; let‐7d‐5p; miR‐20a‐5p; miR‐103a‐3p; miR‐185‐5p; miR‐425‐5p; miR‐4505; miR‐4741			Low let‐7c‐5p, let‐7d‐5p, miR‐20a‐5p, miR‐103a‐3p, miR‐185‐5p,miR‐425‐5p in MM *vs.* HD; low let‐7c‐5p, miR‐20a‐5p, miR‐103a‐3p, miR‐140‐3p, miR‐185‐5p in MM *vs.* SMM; low miR‐20a‐5p, miR‐103a‐3p and high miR‐425‐5p, miR‐4505 in SMM *vs.* HD			(72)
Small EVs	Precipitation (ExoQuick-LP solution for plasma and ExoQuick solution for cell culture supernatants, System Biosciences)	Plasma (0.1 mL), primary MSC from MM patients resistant/sensitive to PI	HMCL	PSMA3; PSMA3-AS1	Increase in the ChT-L activity of the proteasome, regulation of PSMA3 levels	Decreased PI sensitivity, increased proliferation	High in MSC-derived small EVs from PI-resistant patients	Higher plasma levels associated with lower PFS and OS	PSMA3-AS1 siRNA	(62)
Small EVs	Precipitation (ExoQuick solution, System Biosciences) + Filtration	HMCL and primary MM cells	Primary (MGUS) BM Fibroblasts		Overexpression of miR-27b-3p and miR-214-3p by de novo synthesis triggered by the transfer of EV-derived WWC2 protein that regulates the Hippo pathway. Overexpression of fibroblast activation markers α-SMA and FAP.	Increased cell proliferation and inhibition of apoptosis	High in BM-fibroblasts from NDMM *vs.* MGUS; high in BM-fibroblasts from RRMM *vs.* NDMM		Transient transfection with synthetic hsa-miR-27b-3p and hsa-miR-214-3p inhibitors (Exiqon, Woburn, MA, USA)	(56)
Small EVs	miRCURY Exosome Isolation Kit (Exiqon)	Serum (0.25 mL)		lncRNA PRINS			Low (MGUS/MM *vs.* HD)			(43)
Small EVs	Qiagen (method not specified)	BM-MSC from HD and MM patients	HMCL	lncRNA LINC00461	Reduction in miR-15a/miR-16 and overexpression of Bcl2	Increased cell proliferation and inhibition of apoptosis	High in BM-MSC derived from MM *vs.* HD		LINC00461 knockdown	(73)
Large EVs	Serial centrifugation	HMCL and plasma	MSC	miR-103a-3p	Increase in miR-103a-3p and reduced expression of RUNX2	Reduced osteoblast differentiation	High large EVs count in MM *vs.* HD			(74)
Large EVs	Serial Centrifugation	Serum, primary MM cells and HMCL	HUVEC	piRNA-823	Overexpression of VEGF, IL-6, ICAM-1, Bcl2. Inhibition of Caspase-3 activation, downregulation of Bax expression. Increased NO production, decreased ROS production.	Increased proliferation, tube formation, invasion. Inhibition of apoptosis. Tumour growth of xenograft MM in mice	High in large EVs isolated from serum of MM patients *vs.* HD	Correlation with Stage (high in ISS 2 and 3)	piRNA-823 inhibitor	(75)

For each study (listed in rows), the source of EVs (blood plasma, serum or type of cell that secretes EVs and from which EVs have been isolated - donor cells), the EVs isolation method, the expected EVs types (small versus large), the type of recipient (target) cells which uptake EVs and which function is modulated by EVs, are indicated. Identified RNAs, molecular and biological effects in recipient cells together with RNAs expression levels, prognostic significance and potential therapeutic targets are also indicated where applicable.

BM-MSC, mesenchymal stromal cells; HMCL, human myeloma cell lines; HD, healthy donor; MM, multiple myeloma; MGUS, monoclonal gammopathy of undetermined significance; SMM, smouldering multiple myeloma; HUVEC, human umbilical vein endothelial cells; ROS, Reactive oxygen species; NO, nitric oxide; lncRNA, long non-coding RNAs; piRNA, Piwi-interacting RNA; miR, micro-RNA; FIH-1, factor-inhibiting hypoxia-inducible factor 1; FAP, fibroblast-activated protein; ChT-L, chymotrypsin-like; Bcl2, B-cell lymphoma 2; PI, proteaosme inhibitor; Sp1, Specificity Protein 1; ALPL, Alkaline Phosphatase; ISS, international staging system; PFS, progression-free survival; OS, overall survival; βTRC, Beta-Transducin Repeat Containing E3 Ubiquitin; CAF, cancer associated fibroblasts; PSMA3, Proteasome 20S Subunit Alpha 3; RUNX2, RUNX Family Transcription Factor 2; α-SMA, α-smooth muscle actin; WWC2, WWand C2 domain containing2; SDF-1, stromal-derived factor 1; ICAM-1, Intercellular adhesion molecule; VEGF, Vascular endothelial growth factor; CXCL1, C-X-C Motif Chemokine Ligand 1; IL, interleukin; IP-10, Interferon gamma-induced protein 10 (CXCL10); MCP-1, monocyte chemoattractant protein-1; CCL-5, C-C Motif Chemokine Ligand 5; PRINS, Psoriasis Associated Non-Protein Coding RNA Induced By Stress.

### EV-RNA Secreted by MM Cells

De Veirman et al. (68) have described how the transfer of miR-146a contained in small EVs isolated from human MM cell lines (HMCL) to mesenchymal stromal cells (MSC) induced overexpression of miR-146a in the MSC and the secretion of several cytokines and chemokines resulting in enhanced MM cell viability and migration. The authors hypothesized these effects were mediated by the Notch signalling pathway, notion supported by the observation that an inhibitor of the endogenous Notch pathway, DAPT, was able to abrogate the miR-146a-induced increase in MSC cytokine elaboration. Similarly, Cheng et al. (63) observed that small EVs isolated from the HMCL OPM2 harboured high levels of miR-21 and miR-146a. These OPM2-small EVs significantly increased MSC proliferation and induced cancer-associated fibroblasts (CAF) transformation. Consistent with these observations the inhibition of miR-21 or miR-146a reduced these effects of OPM2-small EVs on MSC. These observations confirm that MM cells secrete functional EVs which are up-taken by stromal cells ultimately promoting the formation of a supportive TME. The potential as therapeutic targets is suggested by inhibition or loss of function experimentation.

### EV-RNA Secreted by Stromal Cells

The transfer of small EV-miRNA derived from BM-MSC to MM cells was first described by Roccaro et al. (66). Importantly, the authors observed a distinct miRNA composition for BM-MSC–derived small EVs obtained from healthy donors (HD) when compared to those derived from MM patients, with miR-15a levels found to be significantly higher in HD-EVs. Moreover, a reduction in the proliferation of MM cells when treated with the HD-derived small EVs was observed, suggesting a tumour-suppressive role for the EV-derived miR-15a. Similarly, Umezu et al. (70) demonstrated that BMSC-EVs isolated from MM patients have a distinct profile compared to BMSC-EVs isolated from HD with increased expression of miR-10a, miR-346, and miR-135b. The distinct profile EV-RNA derived from BMSC may aid in the diagnostics workup for MM patients. The authors (70) further observed that transfer of EVs-derived miR-10a to HMCL induced cell proliferation. The latter could be abrogated by an inhibitor of βTRC, the direct target of miR-10a βTRC and by FTY720 (fingolimod), a S1P modulator which inhibits S1P signalling on multivesicular endosomes, thus blocking exosomal multivesicular endosome maturation. This observation further supports the evidence for EVs and EV-RNA as mediators of MM progression and as new therapeutic targets. Deng et al. (73) described an alternative mechanism for induction of MM cell proliferation involving the lncRNA LINC00461 acquired *via* MSC-derived EVs, indicating a functional role not only for miRNA but also for lncRNA in MM.

### EV-RNA and Bone Disease

The formation of osteolytic lesions is a characteristic feature of MM due to increased osteolysis and reduced osteoblastogenesis and underlies many of the clinical signs and symptoms of the disease such as fractures, pain and hypercalcemia (58). The treatment of MM bone disease in addition to the utilisation of anti-MM drugs represents a critical aspect of the management of patients with MM (2, 76–78). A better understanding of the biological processes that lead to bone destruction, therefore, may provide important insights to inform improved management of MM patients.

Several recent reports have described the role of small EV-RNA in MM bone disease supporting the notion that MM is a complex disease with active involvement of osteoclasts and osteoblasts mediated also by EVs. Importantly, these functions can be targeted aiding in the current management of MM-related bone disease. Li et al. (69) identified lncRUNX2-AS1 in EVs derived from HMCL. The lncRNA-RUNX2-AS1 regulates RUNX2 pre-mRNA by blocking its splicing in MSC and the subsequent inhibition of differentiation of MSC is observed. *In vivo* experiments confirmed increased expression of RUNX2 and lower expression of lncRUNX2-AS1 in MSC derived from mice xenografted with HMCL when compared to a control cohort or mice treated with the EV-secretion inhibitor GW4869. Another group made a similar observation with inhibition of osteoblast differentiation in MSC after treatment with MM-derived large EVs associated with an increase in miR-103a-3p levels and inhibition of bone formation *via* RUNX2 targeting (74).

Raimondo et al. (65) demonstrated enrichment of miR-129-5p in both MM-small EVs and SMM-small EVs, more so in the former, identifying miR-129-5p as a possible mediator of EV-induced bone disease. miR-129-5p targets different mRNA involved in osteoblast differentiation, suggesting selective EV-packaging correlated with MM disease stage viz MM versus SMM. The authors demonstrated the transfer of miR-129-5p derived from MM-EVs into MSC resulting in inhibition Sp1 expression, a positive modulator of osteoblastic differentiation, and of its target alkaline phosphatase-ALPL.

### EV-RNA and Angiogenesis

Angiogenesis plays a crucial role in promoting tumour progression (57) with cancer-derived EVs having been shown to enhance the proliferation, migration and tube formation of endothelial cells (ECs). The MM-BM becomes more hypoxic due to the outgrowth of MM cells, promoting their production of greater amounts of small EVs compared to normoxic conditions. Small EVs produced by hypoxic MM cells have been reported to carry the oncogenic miR-135b, which promotes endothelial tube formation by blocking the expression of factor-inhibiting hypoxia-inducible factor 1 (FIH-1) in ECs (67).

The enrichment of MM-derived large EVs with piRNA-823 and their transfer to ECs has been recently described by Li et al. (75). An increase in proliferation, tube formation and invasion along with reduced apoptosis was observed in association with enhanced expression of VEGF, IL-6, and ICAM-1. ECs transfected with piRNA-823 mimic or pre-treated with MM- large EVs were also shown to promote tumour growth in murine MM-xenografts. In contrast, the transfection with a piRNA-823 inhibitor or treatment with EVs from piRNA-823 inhibitor-transfected-MM cells had diametrically opposite effects broadening the scenario for MM combination treatments by specifically targeting EV-mediated angiogenesis.

### EV-RNA and Drug Resistance

Eventually all MM patients develop drug resistance with recent evidence of EV-RNA mediated proteasome inhibitor (PI)-resistance which may be reverted by co-administering specific EV-RNA therapeutics. A unique role of small EVs enriched in lncRNA PSMA3-AS1 in transmitting PI-resistance from MSC to MM cells has recently been described by Xu et al. (62). The authors observed reduced sensitivity of MM cells to the PI bortezomib when treated with MSC-derived EVs isolated from bortezomib-resistant patients. In contrast, MSC-EVs of bortezomib-sensitive patients did not affect the sensitivity of MM cells to PI. PSMA3 and PSMA3-AS1 transcripts were identified in MSC-derived EVs isolated from bortezomib-resistant patients. PSMA3-AS1 is a lncRNA that modulates the levels of PSMA3 which encodes the proteasome type-3 alpha subunit. PSMA3 and PSMA3-AS1 expression levels were upregulated in MM cells treated with MSC-EVs derived from bortezomib-resistant patients. The upregulation of these two transcripts induced proteasome activity, which could explain the resistance to PI. The authors further confirmed these observations *in vivo*. Importantly, PSMA3-AS1 downregulation *via* siRNA increased the sensitivity of HMCL xenografted in mice to PI.

## Translational Applications: EV-RNA as Liquid Biomarker in MM

Few MM studies have performed analyses of EV-RNAs utilising peripheral blood despite evidence suggesting that EVs contain approximately half of all circulating RNAs found in the blood (79). A relationship between EV-RNA levels and patient outcomes has been explored in MM, highlighting the translational potential of EV-RNA and paving the way for its application in MM clinical practice. Several studies have specifically looked at the discriminative potential of EV-RNA between HD and MGUS or MM patients at diagnosis. Caivano et al. suggested the diagnostic potential for small EV-derived miR-155, with serum levels that were found to be significantly lower in MM patients when compared to HD (64). Similarly, EVs derived from the plasma of MGUS or MM patients have significant lower levels of lncRNA PRINS compared to HD (43). Kubiczkova et al. (71) observed that miR-34a and let-7e derived from circulating small EVs can discriminate MGUS and MM from HD with high sensitivity and specificity. Similarly, Zhang et al. (72) demonstrated that serum-derived let‐7c‐5p, let‐7d‐5p, miR‐20a‐5p, miR‐103a‐3p, miR‐185‐5p, miR‐425‐5p levels were significantly lower in small EVs isolated from MM patients when compared to HD, while the levels of miR‐4505 and miR‐4741 were significantly higher. Low levels of circulating small EVs-derived miR‐20a‐5p, miR‐103a‐3p and high levels of miR‐425‐5p, miR‐4505 were also able to discriminate SMM from HD. The authors also showed that low levels of let‐7c‐5p, miR‐20a‐5p, miR‐103a‐3p, miR‐140‐3p, miR‐185‐5p and high levels of miR‐4505 and miR‐4741 differentiated MM-EVs when compared to SMM-EVs.

Circulating small EVs isolated from the serum of 156 patients with MM patients were shown to be enriched in let-7b and miR18a; these two miRNAs have been previously implicated in MM progression (8). Lower levels of both let-7b and miR-18a were significantly associated with poor prognosis (PFS and OS) in newly diagnosed patients treated with bortezomib-containing induction therapy (pre-autologous stem cell transplantation), confirming their predictive value in the context of MM and their utility for improved risk stratification at diagnosis (e.g. patients with poor vs good prognosis or bortezomib resistance vs sensitivity) (8). The source of these circulating EV-miRNA remains unknown as no correlation was found between miRNA from EV or BM MM cells.

Xu et al. (62) have suggested a role for plasma-derived small EV PSMA3 and lncRNA PSMA3-AS1 as prognostic markers with lower levels of small-EVs PSMA3 and PSMA3-AS1 both associated with poorer PFS and OS for newly diagnosed MM patients treated with bortezomib-containing regimens.

## Translational Applications: RNA-Engineered EVs as Therapeutic Strategy in MM

The observation that EVs can transfer molecules (e.g. RNA, proteins) to recipient cells has prompt translational research focused on delivering therapeutic (cytotoxic or inhibitory) payloads with EVs (80). In addition, ligands displayed on the EV surface can engage cell receptors to activate cytosolic signalling pathways in both tumor and microenvironment compartments (81). Despite technical constraints (82), several studies have reported cargo-specific effects when these two strategies are used alone or in combination in cancer with several ongoing clinical trials involving EVs, the vast majority of which applied to solid tumors (83–85).

RNA-armed EVs or engineered EV-like nanoparticles that contain RNA have been successfully utilized in lymphoma models demonstrating for example that siRNA-particles promoted silencing of c-Myc with subsequent activation of poly (ADP-ribose) polymerase-dependent apoptotic pathways in treated λ820 lymphoma cells (86). This may represent a promising tool being MM a Myc-driven cancer. Lipid nanoparticle-based formulations (DCR-MYC) have been used to deliver siRNA into tumor cells, leading to inhibition of translation and expression of the c-Myc protein. This approach was later found to not meet therapeutic expectations (87), however work using antisense oligonucleotides to target c-Myc mRNA continues (88–90). More recently, a shRNA strategy has been used to silence TGF-β1 in lymphoma cells, forcing them to release TGF-β1-depleted EV, thus removing a strong antitumor–immune surveillance inhibitor and increasing the response of the immune system against leukemic cells (91). Loading shRNA into these delivery systems has also the potential to reduce tumor EV production, release, and uptake (81). Furthermore, EVs are able to epigenetically reprogram target cells and to completely change their phenotype in a short time (84) as suggested also by the studies described in this review (75). A potential strategy in the re-activation of the immune system or suppression of osteoclastic activation can be to inhibit the molecules responsible for these deep changes as well as creating engineered EVs carrying specific cargo components (e.g., siRNA and shRNA) that are able to lift the immune cell exhaustion and/or improve bone disease by rewiring the epigenetic landscape. Small EVs may also be a promising delivery platform for CRISPR/Cas9 gene editing for targeted therapies as recently described by McAndrews et al. (92) in pancreatic cancer models.

## Limitations and Technical Challenges

Unfortunately, to date, a lack of standardised methodologies and reporting of results has limited inter-study comparisons and the progression of the translational potential of EVs. Specifically, in MM there is no consensus on the type of starting material (serum versus plasma) and pre-analytical variables are not always reported, e.g. detailed collection procedures and the type of collection tube or anticoagulant which are known to significantly affect the yield and characteristics of EVs. The generation and optimization of methods to isolate EVs and EV-RNA subpopulations with high purity from biological samples, and, to analyse their contents, is a current unmet need in the field. Commercially available kits, proven by us (31) and other groups to provide adequate material with immediate translational implications when compared to time-consuming methods such as the ‘gold standard’ ultracentrifugation, employ a variety of isolation modalities including membrane-base affinity, size exclusion chromatography, precipitation (8, 62, 71). The latter remains the most adopted method in MM despite its inadequacy in removing plasma abundant factors which may affect downstream applications and their interpretation. The sub-optimal methodologies for EV isolation utilized in several MM studies renders the purity of EVs and EV-RNA questionable. Unpublished data from our group demonstrate the importance of samples collection and preparation for EV-RNA analyses. The type of collection tube, the amount of starting material, protocols for RNA isolation and preparation (e.g. DNase treatment), quality control and library preparation for sequencing representing only some of the critical aspects to take into consideration when studying and reporting on EV-RNA. Of critical importance in the study of circulating factors (e.g. EVs) is the demonstration of their source but current methods for isolating EVs from complex biofluids such as blood do not define the cell or tissue of origin. In this context platelet-derived vesicles represent the most abundant of the blood-EVs and specific protocols for platelet-free plasma preparation tailored to minimize the activation and release of platelet-EVs should be widely adopted and accurately reported. The level of platelet EV-markers (e.g. CD41, CD62) may indicate the amount of ‘contamination’ of the EV-sample by platelet-EVs and should be confirmed, for example by immunoblotting as recently described by our group (31). Alternatively, immunocapture has been successfully utilized to exclude this population from EVs of interest (e.g. CD41¯) (15). A potential strategy for defining the derivation of blood EVs would be a side-by-side comparison of EVs with putative source material, for example, cells or tissues such as primary tumours. Omic strategies may provide important insights into specific EV-cargo enrichment and the likely EV-source. In support of this concept a recent report by Hoshino et al. (14) provided a comprehensive analysis of circulating small EVs enriched for unique tumour-related proteomic signatures in solid tumours, confirming that protein packaging reflects tumour biology and is heterogeneous across 16 tumour types. Ultracentrifugation was utilized for EV isolation in the described work and although still considered the ‘gold standard’ for EV studies, it requires high starting volumes, long processing time, specialized equipment, and it has been shown to co-isolate factors that can potentially affect downstream applications and their interpretation making it unsuitable in clinical practice.

## Conclusions and Future Perspectives

Increasing evidence suggests that the interrogation of EVs and EV-RNA may represent a reliable non-invasive and alternative strategy to aid in the diagnosis, prognosis and treatment of solid tumours, highlighted by the recent introduction of EV-based liquid biopsy into clinical practice guidelines for prostate cancer (30). However, advancement in this technology is required to address these needs in other types of cancers, including haematological malignancies and MM with a consensus statement urgently needed tailored for the MM scientific community. The reports mentioned in this review highlight the potential suitability of EVs and EV-RNA as novel factors that could be translated, in the near future, into clinical practice to better manage MM. Referring to publicly available data and analysis tools, conducting studies with a collaborative approach between laboratories, the use of specific liquid biomarkers approaches may also accelerate progress toward EV-standardization and translational application of EVs. Access to larger sample sets from annotated patient cohorts with matched clinical information, including response to therapy and survival, would enable further studies exploring the molecular mechanisms modulating EV-biogenesis, the heterogeneity in EV subtypes, EV targeting and cargo release, the exact composition and characteristics of MM-derived EVs and the physiological relevance of their EV-RNA cargo. This in turn could lead to the identification of novel potential therapeutic targets. Besides providing novel biomarkers and therapeutic targets, the natural nanostructure and modifiable surface properties of exosomes make them a good candidate for drug delivery or immunomodulatory therapy.

Growing evidence suggests a critical role for cell-free (non-EV) nucleic acids, both ctDNA and exRNA, as liquid biomarkers to improve the outcome for MM patients (19). One could envisage that combinatorial liquid biopsy strategies incorporating EVs, cell-free nucleic acids and proteomics/metabolomics may represent a readily accessible and realistic opportunity for improving our understanding and management of the incurable blood cancer MM.

## Author Contributions

AR and AS conceived the project, wrote the initial draft, and finalized the manuscript. SM and TK contributed to modifications of the initial draft. All authors contributed to the article and approved the submitted version.

## Funding

This work was supported by Monash University, Melbourne, Australia – Australian Government Training Program (RTP) scholarship and Monash Departmental Scholarship (AR).

## Conflict of Interest

The authors declare that the research was conducted in the absence of any commercial or financial relationships that could be construed as a potential conflict of interest.

## Publisher’s Note

All claims expressed in this article are solely those of the authors and do not necessarily represent those of their affiliated organizations, or those of the publisher, the editors and the reviewers. Any product that may be evaluated in this article, or claim that may be made by its manufacturer, is not guaranteed or endorsed by the publisher.

## Abbreviations

MM, multiple myeloma; EVs, extracellular vesicles; EV-RNA, extracellular vesicles-derived RNA; PCs, plasma cells; BM, bone marrow; ctDNA, circulating tumour DNA; exRNA, cell-free or extracellular RNA; TME, tumour microenvironment; RBPs, RNA binding proteins; mRNA, messenger RNAs; lncRNA, long non-coding RNAs; miRNA, micro-RNAs; ERCC, Extracellular RNA Communication Consortium; BMME, bone marrow microenvironment; MGUS, monoclonal gammopathy of undetermined significance; SMM, smouldering multiple myeloma; HMCL, human myeloma cell lines; MSC, mesenchymal stromal cells; CAF, cancer associated fibroblasts; FAP, fibroblast-activated protein; α-SMA, α-smooth muscle actin; SDF-1, stromal-derived factor 1; HD, healthy donor; ECs, endothelial cells; FIH-1, factor-inhibiting hypoxia-inducible factor 1; PI, proteasome inhibitor.

## References

[1] Kumar SK Rajkumar V Kyle RA van Duin M Sonneveld P Mateos MV. Multiple Myeloma. Nat Rev Dis Primers (2017) 3:17046. 10.1038/nrdp.2017.46 28726797

[2] Rajkumar SV. Multiple Myeloma: 2020 Update on Diagnosis, Risk-Stratification and Management. Am J Hematol (2020) 95(5):548–67. 10.1002/ajh.25791 32212178

[3] Kumar SK Rajkumar SV. The Multiple Myelomas - Current Concepts in Cytogenetic Classification and Therapy. Nat Rev Clin Oncol (2018) 15(7):409–21. 10.1038/s41571-018-0018-y 29686421

[4] Lakshman A Rajkumar SV Buadi FK Binder M Gertz MA Lacy MQ. Risk Stratification of Smoldering Multiple Myeloma Incorporating Revised IMWG Diagnostic Criteria. Blood Cancer J (2018) 8(6):59. 10.1038/s41408-018-0077-4 29895887 PMC5997745

[5] Lonial S Dhodapkar MV Rajkumar SV. Smoldering Myeloma and the Art of War. J Clin Oncol (2020) 38(21):2363–5. 10.1200/JCO.20.00875 32463739

[6] Mithraprabhu S Spencer A. Liquid Biopsy in Multiple Myeloma. In: Hematology - Latest Research and Clinical Advances. Intechopen (2018). 10.5772/intechopen.72652

[7] Harshman SW Canella A Ciarlariello PD Agarwal K Branson OE Rocci A. Proteomic Characterization of Circulating Extracellular Vesicles Identifies Novel Serum Myeloma Associated Markers. J Proteomics (2016) 136:89–98. 10.1016/j.jprot.2015.12.016 26775013 PMC4783258

[8] Manier S Liu CJ Avet-Loiseau H Park J Shi J Campigotto F. Prognostic Role of Circulating Exosomal miRNAs in Multiple Myeloma. Blood (2017) 129(17):2429–36. 10.1182/blood-2016-09-742296 PMC5409448 28213378

[9] Mithraprabhu S Khong T Ramachandran M Chow A Klarica D Mai L. Circulating Tumour DNA Analysis Demonstrates Spatial Mutational Heterogeneity That Coincides With Disease Relapse in Myeloma. Leukemia (2017) 31(8):1695–705. 10.1038/leu.2016.366 27899805

[10] Mithraprabhu S Sirdesai S Chen M Khong T Spencer A. Circulating Tumour DNA Analysis for Tumour Genome Characterisation and Monitoring Disease Burden in Extramedullary Multiple Myeloma. Int J Mol Sci (2018) 19(7):1858. 10.3390/ijms19071858 PMC6073672 29937522

[11] Chen M Mithraprabhu S Ramachandran M Choi K Khong T Spencer A. Utility of Circulating Cell-Free RNA Analysis for the Characterization of Global Transcriptome Profiles of Multiple Myeloma Patients. Cancers (Basel) (2019) 11(6):887. 10.3390/cancers11060887 PMC6628062 31242667

[12] De Luca L Laurenzana I Trino S Lamorte D Caivano A Musto P. An Update on Extracellular Vesicles in Multiple Myeloma: A Focus on Their Role in Cell-to-Cell Cross-Talk and as Potential Liquid Biopsy Biomarkers. Expert Rev Mol Diagn (2019) 19(3):249–58. 10.1080/14737159.2019.1583103 30782029

[13] Mithraprabhu S Morley R Khong T Kalff A Bergin K Hocking J. Monitoring Tumour Burden and Therapeutic Response Through Analysis of Circulating Tumour DNA and Extracellular RNA in Multiple Myeloma Patients. Leukemia (2019) 33(8):2022–33. 10.1038/s41375-019-0469-x 30992504

[14] Hoshino A Kim HS Bojmar L Gyan KE Cioffi M Hernandez J. Extracellular Vesicle and Particle Biomarkers Define Multiple Human Cancers. Cell (2020) 182(4):1044–61.e18. 10.1016/j.cell.2020.07.009 32795414 PMC7522766

[15] Rajeev Krishnan S De Rubis G Suen H Joshua D Lam Kwan Y Bebawy M. A Liquid Biopsy to Detect Multidrug Resistance and Disease Burden in Multiple Myeloma. Blood Cancer J (2020) 10(3):37. 10.1038/s41408-020-0304-7 32170169 PMC7070076

[16] Reale A Khong T Xu R Savvidou I Lim S Carmichael I. Understanding the Role of Extracellular Vesicles in Multiple Myeloma. In: Copland B, editor. Hematologic Malignancies. Recent Advances in Hematology Research. Nova Science Publishers, Inc (2020). p. 217–61.

[17] Reale A Khong T Xu R Chen M Mithraprabhu S Bingham N. Human Plasma Extracellular Vesicle Isolation and Proteomic Characterization for the Optimization of Liquid Biopsy in Multiple Myeloma. Methods Mol Biol (2021) 2261:151–91. 10.1007/978-1-0716-1186-9_10 33420989

[18] Mithraprabhu S Hocking J Ramachandran M Choi K Klarica D Khong T. DNA-Repair Gene Mutations Are Highly Prevalent in Circulating Tumour DNA From Multiple Myeloma Patients. Cancers (Basel) (2019) 11(7):917. 10.3390/cancers11070917 PMC6678219 31261969

[19] Mithraprabhu S Chen M Savvidou I Reale A Spencer A. Liquid Biopsy: An Evolving Paradigm for the Biological Characterisation of Plasma Cell Disorders. Leukemia (2021). 10.1038/s41375-021-01339-6 34262132

[20] Théry C Witwer KW Aikawa E Alcaraz MJ Anderson JD Andriantsitohaina R. Minimal Information for Studies of Extracellular Vesicles 2018 (MISEV2018): A Position Statement of the International Society for Extracellular Vesicles and Update of the MISEV2014 Guidelines. J Extracell Vesicles (2018) 7(1):1535750. 10.1080/20013078.2018.1535750 30637094 PMC6322352

[21] Xu R Rai A Chen M Suwakulsiri W Greening DW Simpson RJ. Extracellular Vesicles in Cancer - Implications for Future Improvements in Cancer Care. Nat Rev Clin Oncol (2018) 15(10):617–38. 10.1038/s41571-018-0036-9 29795272

[22] Chen M Xu R Rai A Suwakulsiri W Izumikawa K Ishikawa H. Distinct Shed Microvesicle and Exosome microRNA Signatures Reveal Diagnostic Markers for Colorectal Cancer. PloS One (2019) 14(1):e0210003. 10.1371/journal.pone.0210003 30608951 PMC6319712

[23] Greening DW Xu R Gopal SK Rai A Simpson RJ. Proteomic Insights Into Extracellular Vesicle Biology - Defining Exosomes and Shed Microvesicles. Expert Rev Proteomics (2017) 14(1):69–95. 10.1080/14789450.2017.1260450 27838931

[24] Xu R Greening DW Rai A Ji H Simpson RJ. Highly-Purified Exosomes and Shed Microvesicles Isolated From the Human Colon Cancer Cell Line LIM1863 by Sequential Centrifugal Ultrafiltration Are Biochemically and Functionally Distinct. Methods (2015) 87:11–25. 10.1016/j.ymeth.2015.04.008 25890246

[25] Brahmer A Neuberger E Esch-Heisser L Haller N Jorgensen MM Baek R. Platelets, Endothelial Cells and Leukocytes Contribute to the Exercise-Triggered Release of Extracellular Vesicles Into the Circulation. J Extracell Vesicles (2019) 8(1):1615820. 10.1080/20013078.2019.1615820 31191831 PMC6542154

[26] Fendl B Weiss R Fischer MB Spittler A Weber V. Characterization of Extracellular Vesicles in Whole Blood: Influence of Pre-Analytical Parameters and Visualization of Vesicle-Cell Interactions Using Imaging Flow Cytometry. Biochem Biophys Res Commun (2016) 478(1):168–73. 10.1016/j.bbrc.2016.07.073 27444383

[27] Pietrowska M Wlosowicz A Gawin M Widlak P. MS-Based Proteomic Analysis of Serum and Plasma: Problem of High Abundant Components and Lights and Shadows of Albumin Removal. Adv Exp Med Biol (2019) 1073:57–76. 10.1007/978-3-030-12298-0_3 31236839

[28] Hoshino A Costa-Silva B Shen TL Rodrigues G Hashimoto A Tesic Mark M. Tumour Exosome Integrins Determine Organotropic Metastasis. Nature (2015) 527(7578):329–35. 10.1038/nature15756 PMC4788391 26524530

[29] Peinado H Alečković M Lavotshkin S Matei I Costa-Silva B Moreno-Bueno G. Melanoma Exosomes Educate Bone Marrow Progenitor Cells Toward a Pro-Metastatic Phenotype Through MET. Nat Med (2012) 18(6):883–91. 10.1038/nm.2753 PMC3645291 22635005

[30] Tutrone R Donovan MJ Torkler P Tadigotla V McLain T Noerholm M. Clinical Utility of the Exosome Based ExoDx Prostate (IntelliScore) EPI Test in Men Presenting for Initial Biopsy With a PSA 2-10 Ng/Ml. Prostate Cancer Prostatic Dis (2020) 23(4):607–14. 10.1038/s41391-020-0237-z PMC7655505 32382078

[31] Reale A Carmichael I Xu R Mithraprabhu S Khong T Chen M. Human Myeloma Cell- and Plasma-Derived Extracellular Vesicles Contribute to Functional Regulation of Stromal Cells. Proteomics (2021) 21(13-14):e2000119. 10.1002/pmic.202000119 33580572

[32] Srinivasan S Yeri A Cheah PS Chung A Danielson K De Hoff P. Small RNA Sequencing Across Diverse Biofluids Identifies Optimal Methods for exRNA Isolation. Cell (2019) 177(2):446–62.e16. 10.1016/j.cell.2019.03.024 30951671 PMC6557167

[33] Lobb RJ Becker M Wen SW Wong CS Wiegmans AP Leimgruber A. Optimized Exosome Isolation Protocol for Cell Culture Supernatant and Human Plasma. J Extracell Vesicles (2015) 4:27031. 10.3402/jev.v4.27031 26194179 PMC4507751

[34] Muller L Hong CS Stolz DB Watkins SC Whiteside TL. Isolation of Biologically-Active Exosomes From Human Plasma. J Immunol Methods (2014) 411:55–65. 10.1016/j.jim.2014.06.007 24952243 PMC4260336

[35] Smolarz M Pietrowska M Matysiak N Mielańczyk Ł Widłak P. Proteome Profiling of Exosomes Purified From a Small Amount of Human Serum: The Problem of Co-Purified Serum Components. Proteomes (2019) 7(2):18. 10.3390/proteomes7020018 31035355 PMC6630217

[36] Sparrow RL Simpson RJ Greening DW. A Protocol for the Preparation of Cryoprecipitate and Cryo-Depleted Plasma for Proteomic Studies. Methods Mol Biol (2017) 1619:23–30. 10.1007/978-1-4939-7057-5_2 28674874

[37] Cheng L Sharples RA Scicluna BJ Hill AF. Exosomes Provide a Protective and Enriched Source of miRNA for Biomarker Profiling Compared to Intracellular and Cell-Free Blood. J Extracell Vesicles (2014) 3(1):23743. 10.3402/jev.v3.23743 PMC3968297 24683445

[38] Stranska R Gysbrechts L Wouters J Vermeersch P Bloch K Dierickx D. Comparison of Membrane Affinity-Based Method With Size-Exclusion Chromatography for Isolation of Exosome-Like Vesicles From Human Plasma. J Transl Med (2018) 16(1):1. 10.1186/s12967-017-1374-6 29316942 PMC5761138

[39] Tian Y Gong M Hu Y Liu H Zhang W Zhang M. Quality and Efficiency Assessment of Six Extracellular Vesicle Isolation Methods by Nano-Flow Cytometry. J Extracell Vesicles (2020) 9(1):1697028. 10.1080/20013078.2019.1697028 31839906 PMC6896440

[40] Van Deun J Mestdagh P Agostinis P Akay Ö Anand S Anckaert J. EV-TRACK: Transparent Reporting and Centralizing Knowledge in Extracellular Vesicle Research. Nat Methods (2017) 14(3):228–32. 10.1038/nmeth.4185 28245209

[41] Witwer KW Buzás EI Bemis LT Bora A Lässer C Lötvall J. Standardization of Sample Collection, Isolation and Analysis Methods in Extracellular Vesicle Research. J Extracell Vesicles (2013) 2:10.3402/jev.v2i0.20360. 10.3402/jev.v2i0.20360 PMC3760646 24009894

[42] Das S Extracellular RNACC Ansel KM Bitzer M Breakefield XO Charest A. The Extracellular RNA Communication Consortium: Establishing Foundational Knowledge and Technologies for Extracellular RNA Research. Cell (2019) 177(2):231–42. 10.1016/j.cell.2019.03.023 PMC6601620 30951667

[43] Sedlarikova L Bollova B Radova L Brozova L Jarkovsky J Almasi M. Circulating Exosomal Long Noncoding RNA PRINS-First Findings in Monoclonal Gammopathies. Hematol Oncol (2018) 36(5):786–91. 10.1002/hon.2554 PMC6585732 30144133

[44] Hu W Liu C Bi ZY Zhou Q Zhang H Li LL. Comprehensive Landscape of Extracellular Vesicle-Derived RNAs in Cancer Initiation, Progression, Metastasis and Cancer Immunology. Mol Cancer (2020) 19(1):102. 10.1186/s12943-020-01199-1 32503543 PMC7273667

[45] Fabbiano F Corsi J Gurrieri E Trevisan C Notarangelo M D’Agostino VG. RNA Packaging Into Extracellular Vesicles: An Orchestra of RNA-Binding Proteins? J Extracell Vesicles (2020) 10(2):e12043. 10.1002/jev2.12043 33391635 PMC7769857

[46] Veziroglu EM Mias GI. Characterizing Extracellular Vesicles and Their Diverse RNA Contents. Front Genet (2020) 11:700. 10.3389/fgene.2020.00700 32765582 PMC7379748

[47] Li S Li Y Chen B Zhao J Yu S Tang Y. Exorbase: A Database of circRNA, lncRNA and mRNA in Human Blood Exosomes. Nucleic Acids Res (2018) 46(D1):D106–D12. 10.1093/nar/gkx891 PMC5753357 30053265

[48] Keerthikumar S Chisanga D Ariyaratne D Al Saffar H Anand S Zhao K. ExoCarta: A Web-Based Compendium of Exosomal Cargo. J Mol Biol (2016) 428(4):688–92. 10.1016/j.jmb.2015.09.019 PMC4783248 26434508

[49] Pathan M Fonseka P Chitti SV Kang T Sanwlani R Van Deun J. Vesiclepedia 2019: A Compendium of RNA, Proteins, Lipids and Metabolites in Extracellular Vesicles. Nucleic Acids Res (2019) 47(D1):D516–D9. 10.1093/nar/gky1029 PMC6323905 30395310

[50] Murillo OD Thistlethwaite W Rozowsky J Subramanian SL Lucero R Shah N. exRNA Atlas Analysis Reveals Distinct Extracellular RNA Cargo Types and Their Carriers Present Across Human Biofluids. Cell (2019) 177(2):463–77.e15. 10.1016/j.cell.2019.02.018 30951672 PMC6616370

[51] Liu T Zhang Q Zhang J Li C Miao YR Lei Q. EVmiRNA: A Database of miRNA Profiling in Extracellular Vesicles. Nucleic Acids Res (2019) 47(D1):D89–93. 10.1093/nar/gky985 PMC6323938 30335161

[52] O’Brien K Breyne K Ughetto S Laurent LC Breakefield XO. RNA Delivery by Extracellular Vesicles in Mammalian Cells and Its Applications. Nat Rev Mol Cell Biol (2020) 21(10):585–606. 10.1038/s41580-020-0251-y 32457507 PMC7249041

[53] Pourhanifeh MH Mahjoubin-Tehran M Shafiee A Hajighadimi S Moradizarmehri S Mirzaei H. MicroRNAs and Exosomes: Small Molecules With Big Actions in Multiple Myeloma Pathogenesis. IUBMB Life (2020) 72(3):314–33. 10.1002/iub.2211 31828868

[54] Mills J Capece M Cocucci E Tessari A Palmieri D. Cancer-Derived Extracellular Vesicle-Associated MicroRNAs in Intercellular Communication: One Cell’s Trash Is Another Cell’s Treasure. Int J Mol Sci (2019) 20(24):6109. 10.3390/ijms20246109 PMC6940802 31817101

[55] Umezu T Imanishi S Azuma K Kobayashi C Yoshizawa S Ohyashiki K. Replenishing Exosomes From Older Bone Marrow Stromal Cells With miR-340 Inhibits Myeloma-Related Angiogenesis. Blood Adv (2017) 1(13):812–23. 10.1182/bloodadvances.2016003251 PMC5727805 29296725

[56] Frassanito MA Desantis V Di Marzo L Craparotta I Beltrame L Marchini S. Bone Marrow Fibroblasts Overexpress miR-27b and miR-214 in Step With Multiple Myeloma Progression, Dependent on Tumour Cell-Derived Exosomes. J Pathol (2019) 247(2):241–53. 10.1002/path.5187 30357841

[57] Vacca A Ria R Reale A Ribatti D. Angiogenesis in Multiple Myeloma. Chem Immunol Allergy (2014) 99:180–96. 10.1159/000353312 24217610

[58] Bingham N Reale A Spencer A. An Evidence-Based Approach to Myeloma Bone Disease. Curr Hematol Malig Rep (2017) 12(2):109–18. 10.1007/s11899-017-0370-5 28243849

[59] Ria R Reale A De Luisi A Ferrucci A Moschetta M Vacca A. Bone Marrow Angiogenesis and Progression in Multiple Myeloma. Am J Blood Res (2011) 1(1):76–89. 22432068 PMC3301416

[60] Ria R Vacca A. Bone Marrow Stromal Cells-Induced Drug Resistance in Multiple Myeloma. Int J Mol Sci (2020) 21(2):613. 10.3390/ijms21020613 PMC7013615 31963513

[61] Raimondo S Saieva L Vicario E Pucci M Toscani D Manno M. Multiple Myeloma-Derived Exosomes Are Enriched of Amphiregulin (AREG) and Activate the Epidermal Growth Factor Pathway in the Bone Microenvironment Leading to Osteoclastogenesis. J Hematol Oncol (2019) 12(1):2. 10.1186/s13045-018-0689-y 30621731 PMC6325886

[62] Xu H Han H Song S Yi N Qian C Qiu Y. Exosome-Transmitted PSMA3 and PSMA3-AS1 Promote Proteasome Inhibitor Resistance in Multiple Myeloma. Clin Cancer Res (2019) 25(6):1923–35. 10.1158/1078-0432.CCR-18-2363 30610101

[63] Cheng Q Li X Liu J Ye Q Chen Y Tan S. Multiple Myeloma-Derived Exosomes Regulate the Functions of Mesenchymal Stem Cells Partially *via* Modulating miR-21 and miR-146a. Stem Cells Int (2017) 2017:9012152. 10.1155/2017/9012152 29333170 PMC5733127

[64] Caivano A La Rocca F Simeon V Girasole M Dinarelli S Laurenzana I. MicroRNA-155 in Serum-Derived Extracellular Vesicles as a Potential Biomarker for Hematologic Malignancies - A Short Report. Cell Oncol (Dordr) (2017) 40(1):97–103. 10.1007/s13402-016-0300-x 27761889 PMC13001561

[65] Raimondo S Urzi O Conigliaro A Bosco GL Parisi S Carlisi M. Extracellular Vesicle microRNAs Contribute to the Osteogenic Inhibition of Mesenchymal Stem Cells in Multiple Myeloma. Cancers (Basel) (2020) 12(2):449. 10.3390/cancers12020449 PMC7072478 32075123

[66] Roccaro AM Sacco A Maiso P Azab AK Tai YT Reagan M. BM Mesenchymal Stromal Cell-Derived Exosomes Facilitate Multiple Myeloma Progression. J Clin Invest (2013) 123(4):1542–55. 10.1172/JCI66517 PMC3613927 23454749

[67] Umezu T Tadokoro H Azuma K Yoshizawa S Ohyashiki K Ohyashiki JH. Exosomal miR-135b Shed From Hypoxic Multiple Myeloma Cells Enhances Angiogenesis by Targeting Factor-Inhibiting HIF-1. Blood (2014) 124(25):3748–57. 10.1182/blood-2014-05-576116 PMC4263983 25320245

[68] De Veirman K Wang J Xu S Leleu X Himpe E Maes K. Induction of miR-146a by Multiple Myeloma Cells in Mesenchymal Stromal Cells Stimulates Their Pro-Tumoral Activity. Cancer Lett (2016) 377(1):17–24. 10.1016/j.canlet.2016.04.024 27102001

[69] Li B Xu H Han H Song S Zhang X Ouyang L. Exosome-Mediated Transfer of Lncrunx2-AS1 From Multiple Myeloma Cells to MSCs Contributes to Osteogenesis. Oncogene (2018) 37(41):5508–19. 10.1038/s41388-018-0359-0 29895968

[70] Umezu T Imanishi S Yoshizawa S Kawana C Ohyashiki JH Ohyashiki K. Induction of Multiple Myeloma Bone Marrow Stromal Cell Apoptosis by Inhibiting Extracellular Vesicle miR-10a Secretion. Blood Adv (2019) 3(21):3228–40. 10.1182/bloodadvances.2019000403 PMC6855114 31698453

[71] Kubiczkova L Kryukov F Slaby O Dementyeva E Jarkovsky J Nekvindova J. Circulating Serum microRNAs as Novel Diagnostic and Prognostic Biomarkers for Multiple Myeloma and Monoclonal Gammopathy of Undetermined Significance. Haematologica (2014) 99(3):511–8. 10.3324/haematol.2013.093500 PMC3943315 24241494

[72] Zhang ZY Li YC Geng CY Wang HJ Chen WM. Potential Relationship Between Clinical Significance and Serum Exosomal miRNAs in Patients With Multiple Myeloma. BioMed Res Int (2019) 2019:1575468. 10.1155/2019/1575468 31915680 PMC6931021

[73] Deng M Yuan H Liu S Hu Z Xiao H. Exosome-Transmitted LINC00461 Promotes Multiple Myeloma Cell Proliferation and Suppresses Apoptosis by Modulating microRNA/BCL-2 Expression. Cytotherapy (2019) 21(1):96–106. 10.1016/j.jcyt.2018.10.006 30409700

[74] Zhang L Lei Q Wang H Xu C Liu T Kong F. Tumor-Derived Extracellular Vesicles Inhibit Osteogenesis and Exacerbate Myeloma Bone Disease. Theranostics (2019) 9(1):196–209. 10.7150/thno.27550 30662562 PMC6332790

[75] Li B Hong J Hong M Wang Y Yu T Zang S. piRNA-823 Delivered by Multiple Myeloma-Derived Extracellular Vesicles Promoted Tumorigenesis Through Re-Educating Endothelial Cells in the Tumor Environment. Oncogene (2019) 38(26):5227–38. 10.1038/s41388-019-0788-4 30890754

[76] Palma BD Guasco D Pedrazzoni M Bolzoni M Accardi F Costa F. Osteolytic Lesions, Cytogenetic Features and Bone Marrow Levels of Cytokines and Chemokines in Multiple Myeloma Patients: Role of Chemokine (C-C Motif) Ligand 20. Leukemia (2016) 30(2):409–16. 10.1038/leu.2015.259 26419509

[77] Ria R Reale A Moschetta M Mangialardi G Dammacco F Vacca A. A Retrospective Study of Skeletal and Disease-Free Survival Benefits of Zoledronic Acid Therapy in Patients With Multiple Myeloma Treated With Novel Agents. Int J Clin Exp Med (2013) 6(1):30–8. PMC3515976 23236556

[78] Ria R Reale A Vacca A. Novel Agents and New Therapeutic Approaches for Treatment of Multiple Myeloma. World J Methodol (2014) 4(2):73–90. 10.5662/wjm.v4.i2.73 25332907 PMC4202483

[79] Wei Z Batagov AO Schinelli S Wang J Wang Y El Fatimy R. Coding and Noncoding Landscape of Extracellular RNA Released by Human Glioma Stem Cells. Nat Commun (2017) 8(1):1145. 10.1038/s41467-017-01196-x 29074968 PMC5658400

[80] Desantis V Saltarella I Lamanuzzi A Melaccio A Solimando AG Mariggio MA. MicroRNAs-Based Nano-Strategies as New Therapeutic Approach in Multiple Myeloma to Overcome Disease Progression and Drug Resistance. Int J Mol Sci (2020) 21(9):3084. 10.3390/ijms21093084 PMC7247691 32349317

[81] Gargiulo E Morande PE Largeot A Moussay E Paggetti J. Diagnostic and Therapeutic Potential of Extracellular Vesicles in B-Cell Malignancies. Front Oncol (2020) 10:580874. 10.3389/fonc.2020.580874 33117718 PMC7550802

[82] Dooley K McConnell RE Xu K Lewis ND Haupt S Youniss MR. A Versatile Platform for Generating Engineered Extracellular Vesicles With Defined Therapeutic Properties. Mol Ther (2021) 29(5):1729–43. 10.1016/j.ymthe.2021.01.020 PMC8116569 33484965

[83] Kamerkar S LeBleu VS Sugimoto H Yang S Ruivo CF Melo SA. Exosomes Facilitate Therapeutic Targeting of Oncogenic KRAS in Pancreatic Cancer. Nature (2017) 546(7659):498–503. 10.1038/nature22341 28607485 PMC5538883

[84] Zhang X Zhang H Gu J Zhang J Shi H Qian H. Engineered Extracellular Vesicles for Cancer Therapy. Adv Mater (2021) 33(14):e2005709. 10.1002/adma.202005709 33644908

[85] Zhang H Wang J Ren T Huang Y Liang X Yu Y. Bone Marrow Mesenchymal Stem Cell-Derived Exosomal miR-206 Inhibits Osteosarcoma Progression by Targeting TRA2B. Cancer Lett (2020) 490:54–65. 10.1016/j.canlet.2020.07.008 32682951

[86] Lunavat TR Jang SC Nilsson L Park HT Repiska G Lasser C. RNAi Delivery by Exosome-Mimetic Nanovesicles - Implications for Targeting C-Myc in Cancer. Biomaterials (2016) 102:231–8. 10.1016/j.biomaterials.2016.06.024 27344366

[87] Miller AJ Chang A Cunningham PN. Chronic Microangiopathy Due to DCR-MYC, a Myc-Targeted Short Interfering RNA. Am J Kidney Dis (2020) 75(4):513–6. 10.1053/j.ajkd.2019.09.011 31866228

[88] Whitfield JR Beaulieu ME Soucek L. Strategies to Inhibit Myc and Their Clinical Applicability. Front Cell Dev Biol (2017) 5:10. 10.3389/fcell.2017.00010 28280720 PMC5322154

[89] Dhanasekaran R Park J Yevtodiyenko A Bellovin DI Adam SJ Kd AR. MYC ASO Impedes Tumorigenesis and Elicits Oncogene Addiction in Autochthonous Transgenic Mouse Models of HCC and RCC. Mol Ther Nucleic Acids (2020) 21:850–9. 10.1016/j.omtn.2020.07.008 PMC7452286 32805488

[90] Madden SK de Araujo AD Gerhardt M Fairlie DP Mason JM. Taking the Myc Out of Cancer: Toward Therapeutic Strategies to Directly Inhibit C-Myc. Mol Cancer (2021) 20(1):3. 10.1186/s12943-020-01291-6 33397405 PMC7780693

[91] Huang F Wan J Hu W Hao S. Enhancement of Anti-Leukemia Immunity by Leukemia-Derived Exosomes Via Downregulation of TGF-Beta1 Expression. Cell Physiol Biochem (2017) 44(1):240–54. 10.1159/000484677 29130994

[92] McAndrews KM Xiao F Chronopoulos A LeBleu VS Kugeratski FG Kalluri R. Exosome-Mediated Delivery of CRISPR/Cas9 for Targeting of Oncogenic Kras(G12D) in Pancreatic Cancer. Life Sci Alliance (2021) 4(9):e202000875. 10.26508/lsa.202000875 34282051 PMC8321670

